# An investigation of low-protein diets’ qualification rates and an analysis of their short-term effects for patients with CKD stages 3–5: a single-center retrospective cohort study from China

**DOI:** 10.1007/s11255-022-03390-3

**Published:** 2022-10-31

**Authors:** Xian-long Zhang, Min Zhang, Nuo Lei, Wen-wei Ouyang, Hui-fen Chen, Bei-ni Lao, Yan-min Xu, Fang Tang, Li-zhe Fu, Xu-sheng Liu, Yi-fan Wu

**Affiliations:** 1 Renal Division, The Second Affiliated Hospital of Guangzhou University of Chinese Medicine (Guangdong Provincial Hospital of Chinese Medicine), Guangzhou, Guangdong China; 2 The Second Clinical College, Guangzhou University of Chinese Medicine, Guangzhou, China; 3 Key Unit of Methodology in Clinical Research, The Second Affiliated Hospital of Guangzhou University of Chinese Medicine (Guangdong Provincial Hospital of Chinese Medicine), Guangzhou, China; 4 Chronic Disease Management Outpatient, The Second Affiliated Hospital of Guangzhou University of Chinese Medicine (Guangdong Provincial Hospital of Chinese Medicine), Guangzhou, China

**Keywords:** Chronic kidney disease, Low-protein diet, Nutrition management, Chronic disease management

## Abstract

**Background:**

The feasibility and efficacy of low-protein diets (LPD) treatment in chronic kidney disease (CKD) is controversial. Based on the characteristics of the Chinese diet, we observe the qualification rates and short-term clinical effects of LPD for CKD patients in our center.

**Methods:**

This is a retrospective cohort study. CKD stages 3–5 patients who were regularly followed up 5 times (over 2 years) and treated with LPD were included. We collected clinical data to observe the changes in LPD qualification rates and divided patients into LPD and non-LPD group according to the average dietary protein intake (DPI) of 5 follow-up time points and compared the changes in primary and secondary outcome measures between the two groups.

**Results:**

We analyzed data from 161 eligible CKD stages 3–5 patients. From baseline to the 5th follow-up time point, the LPD qualification rates of all patients were 11.80%, 35.40%, 47.82%, 53.43% and 54.04%, respectively. For primary outcome measures, the urine protein/creatinine ratio (UPCR) decreased more in the LPD group than in the non-LPD group [Median (interquartile range, IQR) of the difference between the 5th follow-up time point and baseline: 0.19 (− 0.01–0.73) vs. 0.10 (− 0.08–0.27), *P* < 0.001]. We constructed three classes of mixed linear models (model I, II, III). The UPCR slopes were all negative in the LPD group and positive in the non-LPD group (*P* < 0.001). Meanwhile, in model I, the estimate glomerular filtration rate(eGFR) decline slope in the LPD group was lower than that in the non-LPD group [slope (standard error): − 1.32 (0.37) vs. − 2.35 (0.33), *P* = 0.036]. For secondary outcome measures, body mass index (BMI) triglycerides (TG), body weight, and fat free mass (FFM) showed stable statistical differences in the comparison of LPD and non-LPD groups, with greater declines in the former.

**Conclusion:**

The results of this study suggest that LPD treatment can reduce UPCR in patients with CKD stages 3–5, and may also delay the decline in eGFR. Meanwhile, it also reduces BMI, TG, body weight, and FFM, thus the need to prevent malnutrition in clinical implementation.

**Supplementary Information:**

The online version contains supplementary material available at 10.1007/s11255-022-03390-3.

## Background

Chronic kidney disease (CKD) has a global prevalence of around 8–16%. It can diminish quality of life, and increase the risk of cardiovascular disease and mortality. As such, it has been recognized as a global public health problem [1, 2]. In recent years, nutrition management has been considered an important approach for conservative treatment for CKD patients, and accordingly, it is gaining attention [3, 4]. Effective nutritional treatment not only can ensure that CKD patients’ nutritional requirements are met, but also can reduce metabolite accumulation, therefore contributing to the control of uremic symptoms and other complications, such as electrolyte disturbances and acid–base imbalances, sodium and water retention, and mineral and bone disorder (CKD-MBD) syndrome [3].

Low-protein diets (LPD) have long been controversial in nutritional management. The initial results of the modification of diet in renal disease (MDRD) study showed that LPD had little benefit in the short-term, and did not delay CKD progression [5]. However, other scholars have arrived at different conclusions after reanalyzing the MDRD study, arguing that inconclusive evidence should not be mistaken for evidence supporting null hypotheses [6]. In 2011, the Clinical Practice Guidelines issued by the British Renal Society (BRS) recommended a protein intake of 0.75 g/kg ideal body weight (IBW)/day for CKD stage 4–5 non-dialysis patients [7]. In 2019, it was changed to 0.8–1.0 g/kg IBW/day, and the BRS declared that they believed there was insufficient evidence to recommend LPD therapy (1C) [8]. In contrast, the 2020 update of the National Kidney Foundation's Kidney Disease Outcomes Quality Initiative (KDOQI) Clinical Practice Guidelines for Nutrition in CKD pointed out that a protein-restricted diet could reduce the risk of end-stage renal disease (ESRD) or death (1A), and improve quality of life (2C). For patients with CKD stages 3–5 and without diabetes, LPD should be limited to 0.55–0.60 g dietary protein/kg body weight (BW)/day, and for patients with CKD stage 3–5 and diabetes, LPD should be limited to 0.60–0.80 g dietary protein/kg BW/day [9].

However, perhaps due to differences in dietary habits, LPD has been recommended for nutritional therapy for CKD in China since the 2005 Consensus on Protein Nutritional Therapy for Chronic Kidney Disease [10]*. The Health Industry Standard of the People's Republic of China—Dietary Guide for Chronic Kidney Disease Patients WS/557–2017* promulgated in 2017 also provides detailed guidance on LPD [11]. The latest *Clinical Practice Guidelines for Nutritional Therapy of Chronic Kidney Disease in China* (2021 Edition) also still recommends protein diet restriction [12].

Another problem pertaining to LPD is that its implementation in clinical settings is difficult. Results from a US survey showed that average daily protein intakes were 1.3 g/kg/day [13]. Thus, to meet LPD treatment standards, it would be necessary to reduce the total protein intake by more than half, which in turn would affect patients' dietary satisfaction and motivation [14]. Coupled with patients’ varying education levels and diet patterns, the lack of effective monitoring and feedback mechanisms in medical institutions, as well as patients’ minimal understanding of nephropathy, insufficient doctor-patient communication, insufficient family support, and other reasons, LPD treatment compliance is often compromised [15]. Moreover, many nephrologists lack training and experience in LPD; they fear malnutrition and are unable to develop detailed LPD regimens. As such, they do not implement the LPD treatment [16].

Our center emphasizes LPD therapy for CKD, and has established a professional nutrition management team to oversee patients’ dietary intake, and to collect clinical data. The Chinese Nutrition Society established the current protein dietary reference intakes (DRIs) in China in 2013, which include the estimated averaged requirement (EAR) and recommended nutrient intake (RNI). The EAR for protein is 60 g/day for adult males and 50 g/day for adult females (0.9 g/kg/day). The RNI for protein is 65 g/day for adult males and 55 g/day for adult females (1.0 g/kg/day). A study showed that the total dietary protein intake of the Chinese population would vary with the seasons, with an average estimate of 68.48 ± 22.07 g/day [17, 18]. However, due to the specificity of the Chinese diet, which consists of primarily mixed foods, there are few international reports on LPD in Chinese CKD patients. With this in mind, how is LPD adherence among CKD patients with Chinese dietary habits? What effect does LPD have on CKD prognosis? Can restricting dietary protein affect nutritional status? We conducted a retrospective study to observe the LPD qualification rates among patients with CKD stages 3–5, based on our data. We then analyzed the short-term effects of LPD on CKD progression.

## Methods

### Study setting and study design

This is a retrospective cohort study. It was conducted in the Chronic Disease Management Center at the Nephrology Department of Guangdong Provincial Hospital of Chinese Medicine, Guangzhou, Guangdong Province, China. This study has been registered on the Chinese Clinical Trial Registry, registration number: ChiCTR1900024633.

### Diagnostic and staging criteria

CKD diagnosis and clinical staging was based on the KDOQI clinical practice guidelines [19]. We estimated GFR using the Chronic Kidney Disease Epidemiology Collaboration equation (CKD-EPI 2009 Serum creatinine) [20].

### Inclusion and exclusion criteria

The inclusion criteria were: (I) patients aged 18 years and older; (II) estimated (e)GFR < 60 mL/min/1.73 m^2^; (III) patients who had signed written informed consent to receive self-management and were willing to share clinical data; (IV) results of routine blood tests, routine urine tests, biochemical tests, urinary protein/creatinine ratio (UPCR), 24-h urine analysis, and body composition analysis needed to have been obtained every 6 months over the previous 2 years; The exclusion criteria were: (I) patients with psychiatric illness or other reasons which prevented their cooperation; (II) patients undergoing renal replacement therapy; (III) Patients with acute, subacute or chronic inflammatory conditions; (IV) patients with cancer; (V) patients who are ingesting steroids.

### Dietary restriction of protein intake

This cohort study’s nutrition management team consisted of 2 nephrologists, 3 nurses, 1 registered dietitian, and several clinical graduate students. After patients had established a complete record file at the chronic disease management center, physicians would prescribe nutritional prescriptions, and nurses would provide dietary education according to the patients’ disease conditions.

For detailed diet management steps, see WS/557–2017 [11]: Step 1: Calculate energy intake and food exchange portion. WS/557–2017 uses the Broca-Katsura formula (Katsura method) to calculate the standard body weight (SBW) for Asians and recommends daily caloric and protein intakes for patients with CKD based on the SBW [21, 22], (male) SBW = (height cm-100) *0.9 (kg); (female) SBW = (height cm-100) *0.9 (kg) -2.5 (kg); for patients with CKD stages 3–5, energy intake needed to be maintained at 35 kcal/kg SBW/day (age ≤ 60 years) or 30 kcal to 35 kcal/kg SBW/day (age > 60 years).

Step 2: Calculate protein intake based on the nephropathy food exchange portion in China. This method, based on the renal exchange list, is a practical tool for dietary planning and facilitates nutritional management of kidney disease in developing countries or regions [23, 24]. It classifies foods into 3 levels according to their protein content: 0–1 g, 4 g, and 7 g. 0–1 g foods included oils, starches, melon vegetables, and some fruits; 4 g foods included grains and yams, green leafy vegetables; 7 g foods included beans, meat, eggs and dairy. Every food portion had a unique weight, but all could provide calories in 90 kcal or multiples of 90 kcal (with the exception of vegetables). The total calories that a patient needed to consume per day divided by 90 kcal was how many food portions that patient needed per day. Not only was this method conducive to rapid LPD calculation, but it also ensured sufficient energy intake.

Step 3: Allocate food. At least 50% of total protein intake needed to be high-quality protein. First, allocate the high-quality protein foods (7 g foods) to guarantee the proportion of high-quality protein, and then allocate the non-high-quality protein food (4 g foods) and consider the rationality of food pairings at the same time. The remaining calories could be provided by the low-protein foods (0–1 g foods) since they contained little or no protein.

Step 4: Develop specific recipes. Pair specific foods according to the results of the above food exchange portion, and according to taste.

### Personalized adjustment

In actual application, the above steps would be fine-tuned based on age, weight, physical activity type, comorbidities, stress conditions, and the specific protein content of the food. As for the intake of minerals and vitamins, we would select appropriate foods according to patient needs. We measured the 24-h urinary urea excretion to assess the patients’ actual protein intake. A professional dietitian from the team would handle any cases with serious conditions or complex nutritional management needs, and nurses would give ordinary patients routine nutritional guidance.

### Assessing nutritional status

We followed up with patients recruited for the study at least every 6 months. At each follow-up, patients would be required to fill out the Subjective Global Assessment (SGA) scale and perform laboratory tests including serum albumin (ALB), triglycerides (TG), total cholesterol (TC), hemoglobin (Hb), and body composition analysis based on bioelectrical impedance.

### Grouping

We measured the 24-h urinary urea excretion to calculate the normalized protein equivalent of nitrogen appearance rate (nPNA) according to the Maroni-Mitch formula. In this way, we could evaluate the patients’ actual protein intake [25]. Next, we calculated the patients’ dietary protein intake (DPI), and then we averaged the DPI of the 5 follow-up time points within 2 years. According to WS/557–2017, we considered patients with DPI < 0.8 g/kg SBW/day qualified, and included them in the LDP group. Otherwise, they were included in the non-LDP group. The specific indicators we used to observe LPD’s effect on kidney-related indicators were as follows.

### Primary and secondary outcome measures

This study’s primary outcome measures were UPCR and estimated glomerular filtration rate (eGFR), where UPCR is a spot (random) urine protein creatinine ratio (P/C ratio), an alternative, rapid and simple method for detecting and estimating quantitative proteinuria assessment with good reliability in a wide range of disease states [26, 27]. The secondary outcome measures were serum creatinine (SCR), blood urea (UREA), serum uric acid (UA), carbon dioxide combining power (CO_2_CP), TG, TC, ALB, Hb and the related indicators of nutrition assessment as measured by a Body Composition Analyzer (Ver. LookinBody120, InBody, South Korea). This included body mass index (BMI), total body water (TBW), extracellular water ratio (EWR), waist-hip ratio (WHR), fat free mass (FFM), arm circumference (AC) and arm muscle circumference (AMC).

We performed all laboratory testing in Guangdong Provincial Hospital of Chinese Medicine’s laboratory, and collected the data from the hospital’s Hospital Information System (HIS).

### Statistical analysis

Continuous variables conforming to a normal distribution are presented as mean ± standard deviation (SD), and we analyzed them with a *t*-test. Continuous variables not conforming to the normal distribution are presented as medians (interquartile range, IQR), and we compared them using a Mann–Whitney *U*-test. Categorical variables are presented as frequencies (percentage), and we compared them using either a chi-square test or Fisher’s exact test. We used mixed linear model (MLM) for repeated measures to calculate the slope changes in the two groups’ indicators. We constructed three classes of random intercepts and random slope models (I, II, III). Then, we entered group, time, and the interaction between group and time into Model I. Model II adjusted the demographic data based on Model I [including age, sex, height, weight, SBW, BMI, education level, retirement status, marital status, comorbidities, systolic blood pressure (SBP) and diastolic blood pressure (DBP)]. We assessed comorbidities using the Charlson comorbidity index, which could predict the risk of death in patients with CKD [28]. Model III adjusted for all covariates in Model II and for body composition analysis indicators (including TBW, EWR, BMI, WHR, FFM, AC and AMC), DPI, dietary energy intake (based on a three-day diet diary) and laboratory test indicators (including SCR, UREA, UA, CO2CP, eGFR, TG, TC, ALB, Hb and UPCR). We used Little's test to validate whether the missing data was a random sample of the total data. We determined that the missing data in this study were indeed randomly missing, and that the missing rate was below 10%. The missing values can be supplemented automatically when using mixed linear model analysis [29]. The study’s significance level was set at *P* ≤ 0.05, and all data was analyzed using SPSS 22.0 (IBM Corp., Armonk, NY, USA).

### Ethical issues

All patients included in the study signed an informed consent that allowed their clinical data to be used for medical research. Additionally, this study was approved by the Ethics Committee at Guangdong Provincial Hospital of Chinese Medicine (Approval notice: AF/04-06.1/10.0, 4, July 2019, ZF2019-153-01).

## Results

### Patient selection and grouping results

A total of 218 patients with CKD stages 3–5 were enrolled from October 01, 2019 to October 31, 2021, and each patient was followed up at least every 6 months. We retrospectively collected clinical data for these 218 patients at 5 follow-up points (2 years). 204 patients completed CKD routine laboratory tests and 189 patients had the results of 24-h urine analysis; 161 patients received regular nutritional status assessment via body composition analyzers. Therefore, 161 patients were eligible for inclusion in the final analysis. Finally, according to the grouping criteria, we included 69 patients from the study in the LPD group, and 92 patients in the non-LPD group.

### Baseline data after grouping

We compared the two groups’ baseline characteristics. Most baseline characteristics showed no significant differences in baseline characteristics. This included demographics, laboratory tests, and body composition analysis between the two groups. However, there were statistically significant differences in height, SBW, primary disease, and DPI between the 2 groups (Tables 1 and 2). The LPD group had higher height, higher SBW, and lower DPI than the non-LPD group. Additionally, patients in the LPD group had a greater proportion of primary glomerulopathy, while patients in the non-LPD group had a greater proportion of diabetic kidney disease and unknown primary disease. In the MLM, we adjusted any of the above variables which had statistically significant differences.

**Table 1:** Baseline characteristics of the demographic data

Baseline characteristics	LPD group *n* = 69	Non-LPD group *n* = 92	*P* value
Age (year)	59.00 (45.50–57.00)	60.00 (51.00–68.00)	0.270*
Sex			0.408^†^
Male	42 (60.90)	50 (54.30)	
Female	29 (39.10)	42 (45.70)	
Height (cm)	164.14 ± 7.85	160.57 ± 8.79	0.008**
Weight (kg)	60.22 ± 10.24	59.82 ± 10.70	0.809**
SBW (kg)	56.75 ± 7.98	53.37 ± 8.91	0.014**
BMI (kg/m^2^)	22.85 (20.62–24.22)	23.35 (20.55–25.08)	0.219*
SBP (mm/Hg)	127.00 (116.50–136.00)	126.00 (118.00–134.00)	0.856*
DBP (mm/Hg)	74.00 (68.00–81.50)	73.00 (66.00–80.00)	0.607*
CCI	4.00 (2.00–5.00)	4.00 (3.00–5.00)	0.333*
CKD stage			0.321^†^
Stage 3	39 (56.50)	60 (65.20)	
Stage 4	19 (27.50)	24 (24.60)	
Stage 5	11 (16.00)	8 (8.70)	
Education level			0.691^‡^
Primary or below	8 (11.60)	12 (13.00)	
Junior high school	15 (21.70)	26 (28.30)	
High school or polytechnic school	21 (30.40)	28 (30.40)	
University or junior college	25 (36.20)	25 (27.20)	
Postgraduate	0	1 (1.10)	
Employment status			0.646^†^
Retired	31 (44.90)	38 (41.30)	
Not retired	38 (55.10)	54 (58.70)	
Marital status			0.499^‡^
Married	64 (92.80)	88 (95.70)	
Unmarried	5 (7.20)	4 (4.30)	
Primary disease			0.010^‡^
Primary glomerulopathy	29 (42.00)	22 (23.90)	
Interstitial nephritis	0	2 (2.20)	
Autoimmune disease	4 (5.80)	1 (1.10)	
Diabetic kidney disease	5 (7.20)	18 (19.60)	
Polycystic kidney disease	6 (8.70)	4 (4.30)	
Nephrosclerosis	19 (27.50)	27 (19.30)	
Obstructive nephropathy	2 (2.90)	3 (3.30)	
Unknown	4 (5.80)	15 (16.30)	

Values are given as *n* (%), mean ± standard deviation (SD), or median (interquartile range, IQR). *P* ≤ 0.05 was considered statistically significant

*SBW* standard body weight, *BMI* body mass index, *SBP* systolic blood pressure, *DBP* diastolic blood pressure, *CCI* Charlson Comorbidity Index, *CKD* chronic kidney disease

*Mann–Whitney *U*-test; **t-test; ^†^Chi-square test; ^‡^Fisher’s exact test

**Table 2:** Baseline characteristics of laboratory tests, dietary protein intake and body composition

Baseline characteristics	LPD group *n* = 69	Non-LPD group *n* = 92	*P* value
SCR (μmol/L)	171.00 (127.00–282.75)	158.50 (120.25–216.00)	0.217*
Urea (μmol/L)	10.56 (7.63–13.58)	9.57 (7.91–12.00)	0.296*
UA (μmol/L)	423.54 ± 81.96	437.92 ± 80.67	0.270**
CO_2_CP (mmol/L)	22.84 ± 3.20	23.36 ± 2.72	0.272**
TG (mmol/L)	1.48 (1.10, 2.14)	1.49 (1.05, 1.93)	0.538*
TC (mmol/L)	4.88 ± 1.14	4.98 ± 1.24	0.598**
eGFR (ml/min/1.73m^2^)	35.49 (19.78–48.08)	37.61 (24.58–46.93)	0.483*
Hb (g/L)	125.13 ± 19.17	124.80 ± 18.16	0.912**
ALB (g/L)	44.91 ± 3.71	44.71 ± 3.47	0.726**
UPCR (g/g)	1.02 (0.22–2.02)	0.56 (0.15–1.52)	0.082*
DPI (g/kg SBW/day)	0.95 (0.83–1.13)	1.08 (0.91–1.23)	0.009*
TBW (kg)	33.10 (27.30–37.85)	31.60 (26.65–36.45)	0.228*
WHR	0.85 ± 0.05	0.85 ± 0.05	0.965**
FFM (kg)	44.85 (37.10–50.98)	43.00 (36.30–49.40)	0.233*
EWR	0.39 (0.38–0.39)	0.39 (0.38–0.39)	0.150*
AC (cm)	28.16 ± 2.63	28.89 ± 2.90	0.109**
AMC (cm)	24.94 ± 2.41	25.21 ± 2.33	0.474**

Values are given as n (%), mean ± standard deviation (SD), or median (interquartile range, IQR). *P* ≤ 0.05 was considered statistically significant

*Mann–Whitney *U*-test; **t-test

*SCR* serum creatinine, *UA* serum uric acid, *CO*_*2*_*CP* carbon dioxide combining power, *TG* triglycerides, *TC* serum total cholesterol, *eGFR* estimated glomerular filtration rate, *Hb* hemoglobin, *ALB* albumin, *UPCR* urinary protein/creatinine ratio, *DPI* dietary protein intake, *TBW* total body water, *WHR* waist-hip ratio, *FFM* fat free mass, *EWR* extracellular water ratio, *AC* arm circumference, *AMC* arm muscle circumference

### DPI and its qualification rate

**Fig. 1:**
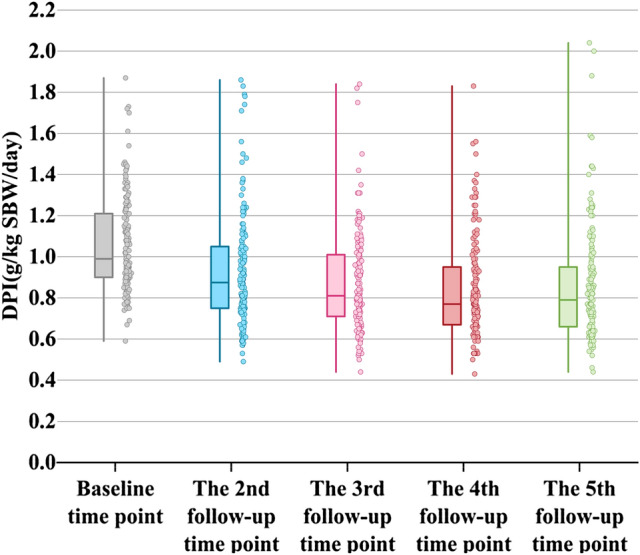
Dietary protein intake for 161 patients at 5 follow-up time points. *DPI* dietary protein intake, *SBW* standard body weight. Each boxplot contains 5 nodes for each dataset, from top to bottom are the maximum value, the 75th percentile, the median, the 25th percentile, and the minimum value. All 161 patients received continuous nutrition education. Most patients had their highest DPI at baseline, and gradually decreased on the 2nd–4th follow-up time points. Compared with the 4th follow-up time point, some patients’ DPI at the 5th follow-up time point increased slightly

**Fig. 2:**
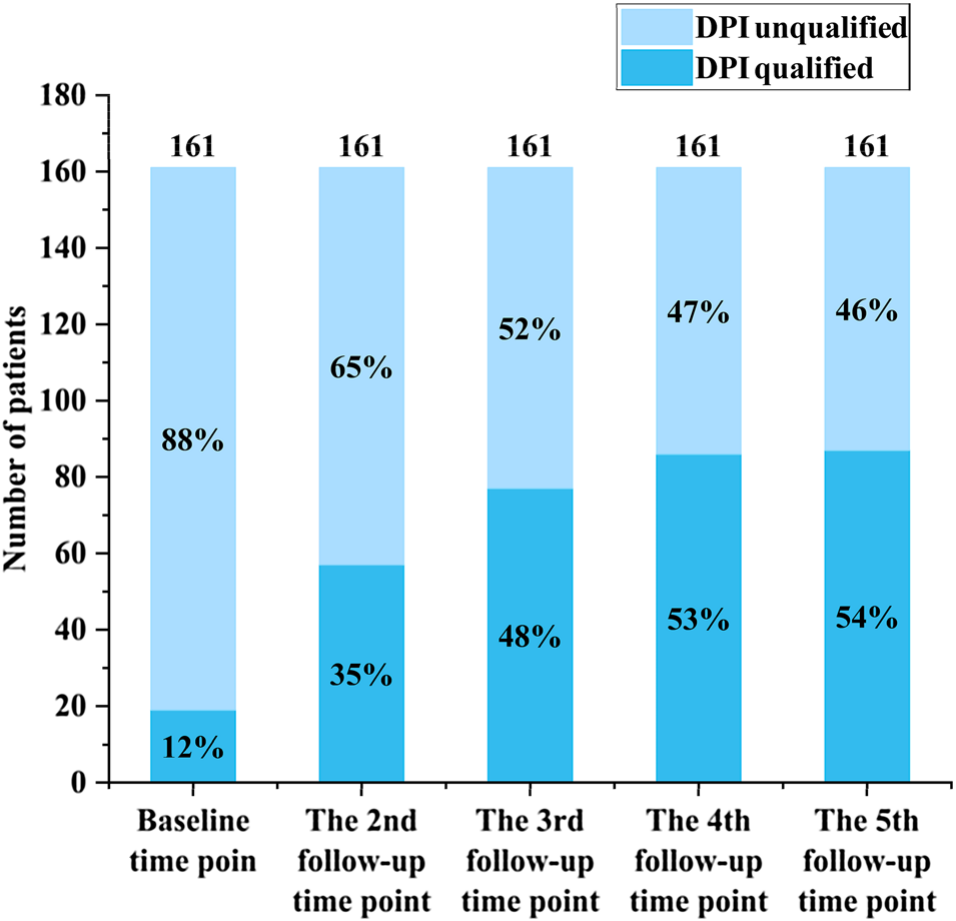
Qualified dietary protein intake rate among 161 patients at 5 follow-up time points. *DPI* dietary protein intake. From baseline to the 5th follow-up time point, the 161 patients’ DPI qualification rate gradually increased and the disqualification rate gradually decreased. The highest increase in qualification rate was at the second follow-up time point, while the lowest increase in qualification rate was at the 5th follow-up time point

Compared with the baseline, most patients’ DPI declined gradually from the second follow-up time point onward, and the qualification rate also gradually increased (Figs. 1 and 2). The median DPI for all patients decreased from 0.99 (0.90–1.21) g/kg SBW/day to 0.77 (0.67–0.95) g/kg SBW/day from baseline to the fourth follow-up time point, with only a slight rebound at the fifth follow-up time point. The median DPI for the fifth follow-up time point was 0.79 (0.66–0.96) g/kg SBW/day. The DPI qualification rate showed a trend of gradual improvement. The DPI qualification rate was 11.80% at baseline and 54.40% at the fifth follow-up point. Meanwhile, DPI in the two patient groups gradually decreased along ensuing follow-up time points. However, DPI decreased more in the LPD group than in the non-LPD group [0.95 (0.83–1.13) to 0.66 (0.61, 0.73) vs. 1.08 (0.93–1.23) to 0.92 (0.82–1.13)]. Among patients with qualified DPI intake at each follow-up time point, the LPD group accounted for a greater proportion than the non-LPD group (Table 3 and Supplementary material, Table 1).

**Table 3:** DPI at 5 follow-up time points

Follow-up time point	DPI *n* = 161	DPI (LPD group) *n* = 69	DPI (Non-LPD group) *n* = 92
Baseline time point	0.99 (0.90–1.21)	0.95 (0.83–1.13)	1.08 (0.93–1.23)
Second follow-up time point	0.87 (0.75–1.05)	0.74 (0.63–0.80)	1.03 (0.89–1.22)
Third follow-up time point	0.81 (0.71–1.01)	0.69 (0.62–0.77)	0.97 (0.83–1.10)
Fourth follow-up time point	0.77 (0.67–0.95)	0.66 (0.62–0.74)	0.93 (0.80–1.12)
Fifth follow-up time point	0.79 (0.66–0.96)	0.66 (0.61–0.73)	0.92 (0.82–1.13)

Values are given as medians (interquartile range, IQR)

*DPI* dietary protein intake

### Primary outcomes

There was no occurrence of renal replacement therapy in this study, from baseline to the 5th follow-up time point. For the primary outcome measures, at the 5th follow-up point, the UPCR of the LPD group was lower than that of the non-LPD group [0.52 (0.23–0.97) vs. 0.95 (0.22–2.00), *P* = 0.050], and from baseline to the 5th follow-up time point, UPCR decline in the LPD group exceeded that of the non-LPD group [0.19 (− 0.01–0.73) vs. − 0.11 (− 0.05–0.03), *P* < 0.001]. Meanwhile, there was no statistically significant difference in eGFR between the two groups at the fifth follow-up time point. There was also no statistically significant difference between the two groups in the difference in eGFR between the baseline and the 5th follow-up time point (Supplementary material, Table 2).

The data obtained in this study were had been measured repeatedly. We used a mixed linear model to compare the differences with regards to various indicators’ trends between the two groups. In this study, we set various indicators (BMI, SBP, DBP, Hb, PCR, ALB, UREA, SCR, CO_2_CP, UA, TG, TC, eGFR, TBW, WHR, FFM, EWR, AC and AMC) as outcome variables, and estimated the varied intercept differences and varied slope differences of various outcome variables over time. Therefore, according to the factors and covariates, 3 classes of models (I, II, and III) were fitted, and models II and III adjusted for factors that had statistically significant differences at baseline. The models’ accuracy could be determined by comparing the − 2log-likelihood. The smaller the value, the more errors were explained, the more reliable the estimated results, and the more accurate the models; the − 2log-likelihoods of most indicators in Model III were superior to those of Model II; Model II also was superior to Model I (supplementary materials). The differences in the variation trend for each indicator could be determined by comparing the slope of the curve. The positive and negative values of the slope indicated the upward and downward varied trend for each indicator, while the magnitude of the slope indicated the degree of the varied trend.

**Fig. 3:**
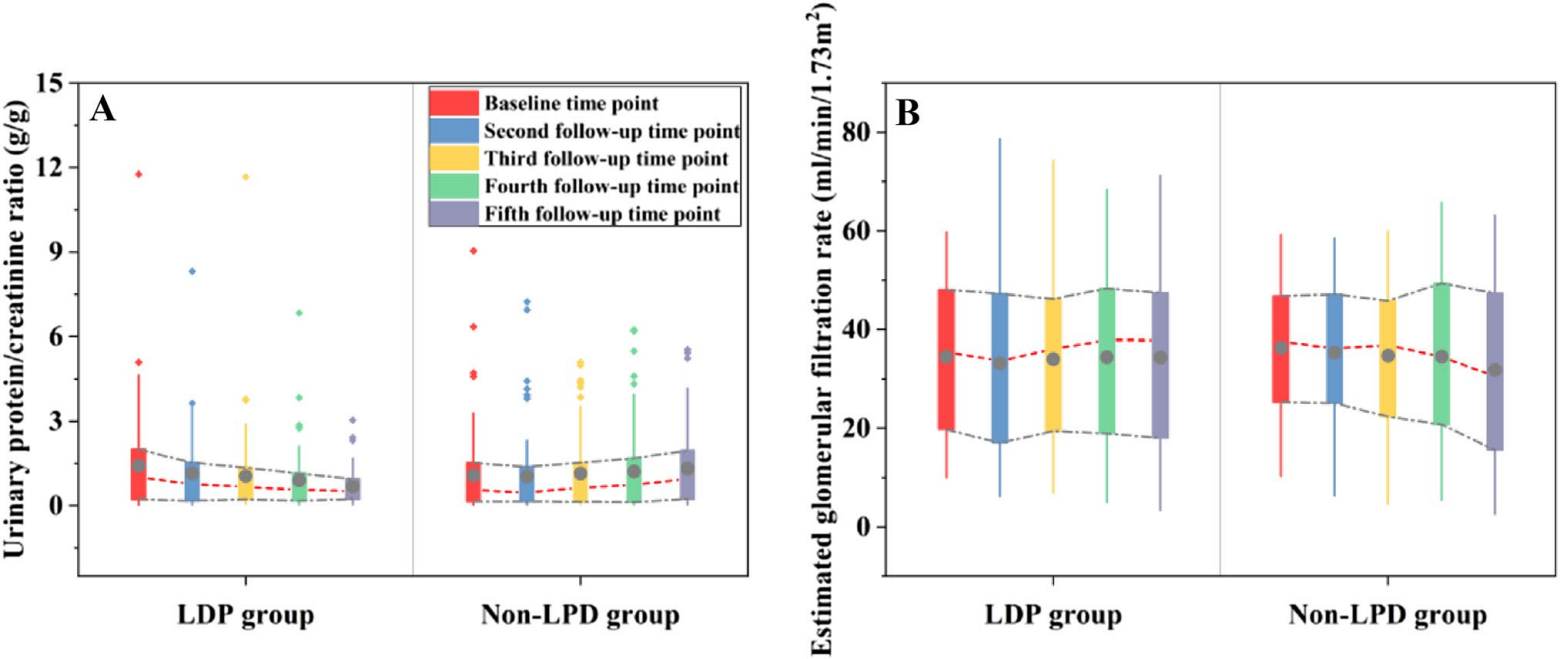
Boxplot of primary outcome measures at 5 follow-up time points. In the boxplot, some data are reflected as outliers. The circles in the middle of the box with the gray fill color are the averages, while the short red underlined lines connect the medians. The 75%th and 25%th quartiles of the five box data are connected by gray line segments, and they form a trend graph over time with the median connecting line. In the primary outcome measures, the urinary protein/creatinine ratio gradually decreased in the LDP group, while the opposite was true for the Non-LPD group. Estimated glomerular filtration rate also showed an opposite trend in both groups, but it was not significant enough

In all three MLMs, the UPCR slopes in the LPD group were negative, presenting an overall downward trend; the UPCR slopes in the non-LPD group were all positive, presenting an overall upward trend [Model I: Slope (standard error, SE): − 0.21 (0.05) vs. 0.17 (0.04), *P* < 0.001. Model II: − 0.21 (0.05) vs. 0.14 (0.05), *P* < 0.001. Model III: − 0.32 (0.06) vs. 0.003 (0.05), *P* < 0.001]. In Model I, there was a statistically significant difference in slope between the two groups, and the decrease in eGFR in the LPD group was less than that in the non-LPD group [− 1.32 (0.37) vs. − 2.35 (0.33), *P* = 0.036]. In Models II and III, there was no statistically significant difference in slope between the two groups (Supplementary material, Table 2). The data distribution trends of the primary outcome measures for the 5 follow-up time points are shown in Fig. 3.

### Secondary outcomes

We compared secondary outcome measures in the LPD and Non-LPD groups, and the trends in the data at the 5 follow-up time points are shown in Supplemental Figs. 1–4. Specific data comparing the two groups are shown in Supplemental Tables 3–6. In terms of renal function, there were no significant differences in SCR, urea, UA or CO_2_CP between the two groups. In terms of lipids, TG and TC were lower in the LPD group than in the non-LPD group, but only TG had a statistically difference between the two groups, as well as in the comparison of Model I and Model II [difference comparison: 0.11 (− 0.29–0.50) vs. − 0.09 (− 0.55–0.28), *P* = 0.035. Model I: − 0.12 (0.05) vs. 0.09 (0.05), *P* = 0.006. Model II: − 0.09 (0.06) vs. 0.08 (0.05), *P* = 0.024]. Comparison of body composition analysis between the two groups showed that in terms of body weight, the LPD group had lower body weight than the non-LPD group, and the LPD group lost more weight compared to the baseline [difference comparison: 1.69 (− 0.41–4.26) vs. 0.69 (− 0.96–2.55), *P* = 0.050]. The BMI of the two patient groups showed a downward trend during the follow-up period, while BMI showed an even greater decrease in the LPD group. [5th follow-up time point: 21.65 (19.58–23.30) vs. 22.40 (20.45–24.35)), *P* = 0.035. Difference comparison: 0.75 (− 0.07–1.57) vs. 0.27 (− 0.39–0.95), *P* = 0.041. Model I: − 0.32 (0.06) vs. − 0.15 (0.05), *P* = 0.032. Model II: − 0.17 (0.04) vs. − 0.04 (0.03), *P* = 0.013. Model III: − 0.17 (0.05) vs. − 0.04 (0.04), *P* = 0.032].

The TBW loss in the LPD group was greater than that in the non-LPD group, but the observed change trend was unstable when we used the MLM to evaluate it [Difference comparison: 0.55 (0–1.28) vs. 0 (− 0.60–0.90), *P* = 0.026. Model I: − 0.25 (0.07) vs. − 0.06 (0.06), *P* = 0.039. Model II: − 0.14 (0.07) vs. 0.06 (0.06), *P* = 0.013. Model III: 0.02 (0.005) vs. − 0.001 (0.004) *P* = 0.003]. During the follow-up period, there was a statistically significant difference between the two patient groups’ WHR slopes, but because the slope value was too small, this might not have had clinical significance [Model I: 0.001 (0.002) vs. − 0.004 (0.001), *P* = 0.026. Model II: 0.003 (0.002) vs. − 0.003 (0.002), *P* = 0.007]. The decrease in FFM in the LPD group was greater than that in the non-LPD group, and all three classes models suggested a downward trend in the LPD group during the follow-up period, while in Models II and III, the non-LPD group showed an upward trend [difference: 0.75 (0–1.78) vs. 0.10 (− 0.80–1.20), *P* = 0.018. Model I: − 0.36 (0.10) vs. − 0.07 (0.08), *P* = 0.025. Model II: − 0.20 (0.09) vs. 0.10 (0.08), *P* = 0.008. Model III: − 0.02 (0.007) vs. 0.003 (0.006), *P* = 0.003]. In addition, the output from Model III suggested that the changing Hb trend in the LPD group might have been an improvement over that of the non-LPD group [2.15 (0.70) vs. 0.43 (0.59), *P* = 0.035].

## Discussion

In this study, we observed the qualification rate and short-term clinical benefits of LPD for patients at our center with stages 3–5 CKD.

The results showed that the LPD qualification rate was only about 50% at the 5th follow-up time point (the 24th month), which is similar to the results from studies in other countries. An Italian cross-sectional study showed that adherence to dietary prescriptions in children and adolescents was 56% [30]. Another retrospective study from Brazil included 321 non-dialysis CKD patients, 158 of whom had adhered to LPD, and the adherence was 49% [31]. There are many reasons for the low LPD qualification rate. In this study, the proportion of diabetic kidney disease patients in the non-LPD group (19.6%) exceeded that in the LPD group (7.2%) (Table 2). Previous studies have shown that implementing LPD is more complicated when patients have diabetic nephropathy. Blood glucose management is more difficult for them as well, and their adherence to management is lower. Therefore, this may be one of the reasons for the low LPD qualification rate in the non-LPD group [32, 33].

Meanwhile, according to the characteristics of the patients in the center, we considered that the reasons for the low qualification LPD rate may have included: (1) The LPD implementation process was indeed cumbersome; (2) The patients in our study were middle-aged or elderly, and the dietary structure was difficult to change; (3) cooking methods and food-pairing for Chinese cuisine is rich and varied, and it is not easy for patients to follow an LPD alone when they are accustomed to eating with family. Therefore, clinicians could improve CKD patients’ low LPD qualification rates in two ways. First, for patients of means, low-protein or protein-free products could be recommended, and energy supplements and keto acid preparations should be taken as needed [34, 35]. Second, personalized nutrition prescriptions could be formulated based on patients' dietary habits and disease conditions. In China, low-protein foods are recommended for CKD patients, including wheat starches, sweet potato starches, cassava starches, mung beans, pea starches and products derived from them; recipes could also be recommended according to patients’ unique situations, to diversify diets. This would not only meet patients’ tastes, but also increase the diversity of patient choices, thereby improving their compliance with LPD [11]. However, the ideal situation would be for patients need to keep in regular communication with the clinical team. Throughout chronic disease management, patients’ dietary habits and dietary structure would gradually approach the LPD standards due to continuous precise nutritional guidance and dietary education. This could improve the compliance of patients with CKD stages 3–5 to LPD [36]. In addition, there is still a lack of reports on whether the nutrient intake qualification rate for patients receiving dietary guidance has continued to improve, or has reversed [37]. The results of this study showed that the DPI qualification rate of 161 patients increased from 11.80% at baseline to 54.40% at the 5th follow-up time point. Therefore, we believe that although the dietary structure in China is complex, LPD is still feasible, and also would optimize the diet of CKD patients through better management.

The results of this study also indicate that LPD could offer short-term benefits for patients with CKD stages 3–5. We performed statistical analysis using a parametric test, a non-parametric test, and a mixed linear model. The primary outcome measure results showed that LPD could reduce urinary protein excretion, reduce UPCR in CKD stages 3–5 patients, and may also slow eGFR decline. The secondary outcome measures showed that LPD reduced TG and decreased body weight, BMI and FFM; it may also have an effect on WHR, TBW and Hb.

CKD patients in Japan, another country with East Asian cultural roots, have also been treated with LPD under comprehensive management. Based on the pooled results of meta-analyses from several clinical studies, Japanese scholars also believed that LPD could reduce urinary protein excretion, protect renal function and alleviate subjective symptoms [38]. The mechanism through which LPD reduces urine protein excretion may be a factor in inhibiting the renin-angiotensin system (RAS) [39]. In clinical practice, the application of angiotensin-converting enzyme inhibitors (ACE-Is) or angiotensin receptor blockers (ARBs) reduces proteinuria, and delays CKD progression in patients with large amounts of proteinuria. Studies have also shown that the overexpression of transforming growth factor-β (TGF-β) influences CKD progression and proteinuria occurrence, and that the combined treatment of ACEI/ARB and LPD reduces TGF-βexpression [40, 41].

The results of this study also suggest that LPD both causes weight loss, and lowers BMI and TG. Obesity and hyperlipidemia are the most prevalent independent risk factors for CKD [42]. Meanwhile, high BMI and lipid metabolism disorders were both risk factors for CKD co-occurring with cardiovascular disease [43]. A previous study has revealed that kidney disease progression is correlated with obesity and high BMI (> 30 kg/m^2^), and has a *U*-shaped relationship with mortality [44]. In addition to body weight, a cross-sectional study in China has also shown that hypertriglyceridemia is correlated with increased urinary albumin-to-creatinine ratios (ACR) [45]. The results of the meta-analysis also showed that LPD could reduce proteinuria, cause weight loss, adjust lipid metabolism, and reduce the onset of azotemia, thereby delaying the patient’s time to end-stage renal disease [46, 47]. It is important to note the “obesity paradox” in CKD patients: a study has shown that obesity is correlated with a lower risk of death in patients with CKD, especially ESRD [48]. This is because obesity may represent the body's nutritional reserves, and studies have suggested that adipose tissue may help chelate uremia toxins in CKD patients [49]. Therefore, in nutrient management, we need to distinguish between the pathological state of abnormal lipid accumulation and the good nutritional state of body fullness, according to patients’ varying conditions, and differentiate between the effects of lipid packing, dyslipidemia, and obesity on patients during different periods. We also must avoid abnormal lipid distribution, lipid loss, and muscle consumption, and provide the most beneficial nutritional guidance for patients [50, 51].

LPD is a safe treatment for CKD patients; it causes few adverse reactions, and few studies have reported protein-energy waste (PEW) during LPD [46, 52]. There were no cases of PEW in this study, which may be related to the absence of patients included in hemodialysis or peritoneal dialysis. In addition, we effectively avoided PEW by several methods, including (I) prescribing nutritional supplements such as keto acids to appropriate patients; (II) recommending low-protein staple foods to increase dietary energy intake; (III) providing ongoing nutritional counseling to optimize dietary nutritional intake; (IV) enhancing management of chronic kidney disease comorbidities; and (V) instructing patients to perform appropriate exercise. Such LPD treatment also does not increase the phosphorus burden, and the data at the beginning and end of this study showed that the median serum phosphorus of patients was about 1.2 mmol/L. At the 5th follow-up time point, we observed no statistically significant differences between ALB and other nutritional monitoring indicators such as AMC and AC, between the two groups. However, the results of the comparison of the difference between the two patient groups and the MLM in this study suggested that during the LPD implementation, patients tended to decrease their body weight and FFM. Although the decline was not large, it still reminds us that we should regularly monitor patient nutrition, follow patients’ nutritional status, and adjust nutrition programs in real-time according to actual situations, so as to prevent lipid loss and muscle waste.

This study has its weaknesses. In addition to the common limitations of non-randomized methods, the follow-up period was not long enough. Moreover, this was a single-center retrospective study, and we did not investigate the specific reasons for LPD non-compliance. We were also unable to perform Kaplan-Mayer and Cox analysis of the primary outcome indicators because no patients had entered the endpoint event by the time data collection was complete. To ensure the integrity of the 24-h urinary urea excretion results and other data, the patients included in the study were screened. Therefore, the sample size of this cohort is small, the patients are younger, and there are fewer comorbidities, and their follow-up adherence may have been better than patients who were not included. The results and limitations of this study highlight the need for further study, and we hope to further verify the therapeutic effects of LPD with prospective and/or randomized studies in the future.

## Conclusion

The results of this study suggest that LPD treatment can reduce UPCR in patients with CKD stage 3–5, and may delay the decline in eGFR. Meanwhile, it also reduces BMI, TG, body weight, and FFM. We found no adverse reactions during LPD implementation, yet we still recommend regular nutritional status assessment to prevent PEW. However, with Chinese dietary habits, the LPD qualification rate for patients with CKD stages 3–5 is still low. Yet guidance and education offer room for additional improvement, and thus, further studies are needed to improve LPD qualification rates according to the characteristics of the Chinese diet.

## Supplementary Information

Below is the link to the electronic supplementary material.

Supplementary file1 (DOCX 597 KB)

## Data Availability

Data used and/or analyzed in the current study are available upon reasonable request to the corresponding author.
