# Supplementary material for: An investigation of low-protein diets’ qualification rates and an analysis of their short-term effects for patients with CKD stages 3–5: a single-center retrospective cohort study from China
Source: Int Urol Nephrol. 2022 Oct 31;55(4):1059–70. doi: 10.1007/s11255-022-03390-3 (PMC10030416; doi:10.1007/s11255-022-03390-3)
Supplement: Supplementary file 1 — Supplementary file1 (DOCX 597 KB) [file 11255_2022_3390_MOESM1_ESM.docx]

**Supplementary materials**

Supplemental Table 1: DPI qualification rate at 5 follow-up points^a^

| **Follow-up time point** | **DPI qualified rate**  **n=161** | **DPI qualified rate**  **(LPD group, n=69)** | **DPI qualified rate**  **(Non-LPD group, n=92)** |
| --- | --- | --- | --- |
| Baseline time point, n (%) | 19 (11.80) | 15 (21.74) | 4 (4.35) |
| Second follow-up time point, n (%) | 57 (35.40) | 52 (75.36) | 5 (5.43) |
| Third follow-up time point, n(%) | 77 (47.82) | 56 (81.16) | 21 (22.83) |
| Fourth follow-up time point, n(%) | 86 (53. 42) | 64 (92.75) | 22 (23.91) |
| Fifth follow-up time(%) | 87 (54.04) | 68 (98.55) | 19 (20.65) |

^a^Values are given as n (%). DPI: dietary protein intake

In this study, we retrospectively analyzed data from 161 patients (69 in an LPD group and 92 in a non-LPD group) at 5 follow-up time points (over 2 years), which were repeated measurements. Therefore, to observe whether each outcome measure had changed over time, we applied a mixed linear model (MLM) for statistical analysis. The MLM was able to estimate the intercept difference and slope difference between the two patient groups for each outcome measure over time. Therefore, if the interaction term (slope) between group and time in the model was statistically significant, this suggested a different trend in the outcome indicators over time between the LPD and non-LPD groups.

We fitted three models (Models Ⅰ, Ⅱ, and Ⅲ) based on different factors and covariates, and the models’ accuracy could be determined by comparing the -2log-likelihood; the smaller the value, the more of the error was explained and the more accurate the model was. Differences in trends in outcome measures between the two groups could be observed by comparing the overall slopes. The slopes’ positive and negative values indicate the curve’s overall upward and downward trend, and the slope’s magnitude indicates the extent of this trend.

Supplementary Tables 2 and 3 present the slopes of each outcome measure for the two groups of patients in the three models. As shown in Supplementary Tables 4-6, model III had the best model accuracy in this study, followed by model II and finally model I.

Supplemental Table 2: The fifth follow-up time point (2 years) of the primary outcome measures, the difference from the baseline, and the results of the mixed linear model^a^

| **Variables**  **LPD group**  **(n=69)**  **Non-LPD**  **group (n=92)** | **Fifth follow-**  **up time point** | ***P* value** | **Difference value^b^** | ***P* value** | **Mixed linear model^c^** | | | | | |
| --- | --- | --- | --- | --- | --- | --- | --- | --- | --- | --- |
|  |  |  |  |  | **Model Ⅰ** | | **Model Ⅱ** | | **Model Ⅲ** | |
|  |  |  |  |  | **Slope**  **(SE)** | ***P***  **value** | **Slope**  **(SE)** | ***P***  **value** | **Slope**  **(SE)** | ***P***  **value** |
| UPCR (g/g) |  |  |  |  |  |  |  |  |  |  |
| LPD group | 0.52  (0.23 -  0.97) | 0.050^*^ | 0.19  (-0.01 -  0.73) | <  0.001^*^ | -0.21  (0.05) | <  0.001 | -0.21  (0.05) | <  0.001 | -0.32  (0.06) | <  0.001 |
| Non-LPD  group | 0.95  (0.22 -  2.00) |  | -0.11  (-0.55 -  0.03) |  | 0.17  (0.04) |  | 0.14  (0.05) |  | 0.003  (0.05) |  |
| eGFR (ml/min/1.73m^2^) |  |  |  |  |  |  |  |  |  |  |
| LPD group | 37.84  (18.04 - 47.42) | 0.494^*^ | -2.05  (-5.78 -  2.97) | 0.076^*^ | -1.32  (0.37) | 0.036 | -1.90  (0.43) | 0.115 | 0.30  (0.39) | 0.325 |
| Non-LPD  group | 30.56  (15.38 - 47.40) |  | -3.86  (-8.47 -  0.52) |  | -2.35  (0.33) |  | -2.70  (0.39) |  | -0.14  (0.32) |  |

^a^Values are given as median (interquartile range, IQR). *P*≤0.05 was considered statistically significant.

^b^Difference value between baseline and the fifth follow-up time point

^c^Mixed linear model of all data at five follow-up time points. Model Ⅰ adjusted for grouping, time, and interactions between groups and time. Model Ⅱ adjusted demographic data on the basis of Model Ⅰ [including age, sex, height, weight, standard body weight (SBW), body mass index (BMI), education level, retirement status, marital status, comorbidities, systolic blood pressure (SBP) and diastolic blood pressure (DBP)]. Model Ⅲ adjusted for all covariates in Model II and body composition analyses, dietary protein intake (DPI), dietary energy intake (DEI) and laboratory tests [including total body weight (TBW), extracellular water ratio (EWR), waist-hip ratio (WHR), fat free mass (FFM), arm circumference (AC), arm muscle circumference (AMC), serum creatinine (SCR), urea, serum uric acid (UA), carbon dioxide combining power (CO_2_CP), estimated glomerular filtration rate (eGFR), triglycerides (TG), total cholesterol (TC), albumin (ALB), hemoglobin (Hb) and urinary protein/creatinine ratio (UPCR)].

SE: standard error, ^*^Mann-Whitney U-test.

Supplemental Table 3: The fifth follow-up time point (2 years) of the secondary outcome measures, the difference from the baseline, and the results of the mixed linear model^a^

| **Variables**  **LPD**  **group**  **(n=69)**  **Non-LPD**  **group**  **(n=92)** | **The fifth follow-**  **up time point** | ***P* value** | **Difference value^b^** | ***P***  **value** | **Mixed linear model^c^** | | | | | |
| --- | --- | --- | --- | --- | --- | --- | --- | --- | --- | --- |
|  |  |  |  |  | **Model Ⅰ** | | **Model Ⅱ** | | **Model Ⅲ** | |
|  |  |  |  |  | **Slope**  **(SE)** | ***P***  **value** | **Slope**  **(SE)** | ***P***  **value** | **Slope**  **(SE)** | ***P***  **value** |
| SCR (μmol/L) |  |  |  |  |  |  |  |  |  |  |
| LPD  group | 150.00  (123.00 - 290.00) | 0.694^*^ | -4.00  (-34.50 - 8.50) | 0.108^*^ | 24.85  (5.61) | 0.537 | 29.32  (6.02) | 0.733 | 9.52  (2.74) | 0.085 |
| Non-LPD  group | 185.00  (124.00 - 324.25) |  | -17.50  (-75.25 - 4.25) |  | 29.47  (4.97) |  | 31.94  (5.40) |  | 3.88  (2.28) |  |
| Urea (μmol/L) |  |  |  |  |  |  |  |  |  |  |
| LPD  group | 9.86  (7.27 - 15.29) | 0.623^*^ | -0.50  (-3.13 - 0.64) | 0.490^*^ | 0.95  (0.24) | 0.477 | 1.10  (0.25) | 0.583 | 0.12  (0.19) | 0.663 |
| Non-LPD  group | 10.33  (7.90 - 14.61) |  | -1.07  (-3.64 - 0.39) |  | 1.18  (0.21) |  | 1.28  (0.23) |  | 0.22  (0.16) |  |
| UA (μmol/L) |  |  |  |  |  |  |  |  |  |  |
| LPD  group | 389.00  (333.00 - 459.50) | 0.240^*^ | 11.00  (-40.50 - 92.50) | 0.680^*^ | -10.98  (5.58) | 0.636 | -7.05  (5.57) | 0.901 | -13.56  (6.58) | 0.818 |
| Non-LPD  group | 412.00  (348.00 - 485.50) |  | 6.50  (-41.50 - 87.00) |  | -7.45  (4.94) |  | -6.14  (5.01) |  | -11.74  (5.43) |  |
| CO_2_CP (mmol/L) |  |  |  |  |  |  |  |  |  |  |
| LPD  group | 21.65 ± 2.66 | 0.724^**^ | -1.50  (-2.95 - 0.60) | 0.767^*^ | -0.62  (0.18) | 0.868 | -0.70  (0.19) | 0.992 | -0.55  (0.21) | 0.974 |
| Non-LPD  group | 21.82 ± 2.89 |  | -1.55  (-3.63 - 0.68) |  | -0.66  (0.16) |  | -0.70  (0.17) |  | -0.54  (0.18) |  |
| TG (mmol/L) |  |  |  |  |  |  |  |  |  |  |
| LPD  group | 1.43  (0.97 - 1.86) | 0.397^*^ | 0.11  (-0.29 - 0.50) | 0.027^*^ | -0.12  (0.05) | 0.006 | -0.09  (0.06) | 0.024 | -0.02  (0.06) | 0.122 |
| Non-LPD  group | 1.49  (1.07 - 2.09) |  | -0.09  (-0.55 - 0.28) |  | 0.09  (0.05) |  | 0.08  (0.05) |  | 0.09  (0.05) |  |
| TC (mmol/L) |  |  |  |  |  |  |  |  |  |  |
| LPD  group | 4.66  (3.86 - 5.67) | 0.144^*^ | 0.15  (-0.45 - 0.68) | 0.143^*^ | -0.12  (0.06) | 0.107 | -0.12  (0.06) | 0.196 | -0.04  (0.07) | 0.759 |
| Non-LPD  group | 5.13  (4.28 - 5.69) |  | -0.01  (-0.67 - 0.50) |  | 0.02  (0.06) |  | 0.02  (0.06) |  | -0.01  (0.05) |  |
| Hb (g/L) |  |  |  |  |  |  |  |  |  |  |
| LPD  group | 126.76 ± 21.34 | 0.191^**^ | 0 (-5.00 - 6.00) | 0.237^*^ | -0.82  (0.63) | 0.424 | -0.91  (0.68) | 0.310 | 2.15  (0.70) | 0.035 |
| Non-LPD  group | 121.94 ± 20.47 |  | -2.00  (-11.00 - 3.00) |  | -1.50  (0.57) |  | -1.78  (0.62) |  | 0.43  (0.59) |  |
| ALB (g/L) |  |  |  |  |  |  |  |  |  |  |
| LPD  group | 44.00  (41.70 - 46.70) | 0.691^*^ | 0.10  (-1.93 - 2.80) | 0.888^*^ | -0.74  (0.23) | 0.626 | -0.74  (0.20) | 0.982 | -1.24  (0.23) | 0.089 |
| Non-LPD  group | 44.10  (41.40 - 45.90) |  | 1.05  (-2.10 - 2.68) |  | -0.89  (0.21) |  | -0.74  (0.18) |  | -0.76  (0.19) |  |
| Weight (kg) |  |  |  |  |  |  |  |  |  |  |
| LPD  group | 57.99 ± 9.56 | 0.701^**^ | 1.69  (-0.41 - 4.26) | 0.050^*^ | -0.43  (0.13) | 0.433 | 0.28  (0.10) | 0.026 | 0.01  (0.04) | 0.397 |
| Non-LPD  group | 58.61 ± 10.21 |  | 0.69  (-0.96 - 2.55) |  | -0.29  (0.11) |  | 0.004  (0.09) |  | -0.03  (0.03) |  |
| BMI (kg/m^2^) |  |  |  |  |  |  |  |  |  |  |
| LPD  group | 21.65  (19.58 - 23.30) | 0.035* | 0.75  (-0.07 - 1.57) | 0.041^*^ | -0.32  (0.06) | 0.032 | -0.17  (0.04) | 0.013 | -0.17  (0.05) | 0.032 |
| Non-LPD  group | 22.40  (20.45 - 24.35) |  | 0.27  (-0.39 - 0.95) |  | -0.15  (0.05) |  | -0.04  (0.03) |  | -0.04  (0.04) |  |
| TBW (kg) |  |  |  |  |  |  |  |  |  |  |
| LPD  group | 31.98 ± 5.99 | 0.460^**^ | 0.55 (0 - 1.28) | 0.026^*^ | -0.25  (0.07) | 0.039 | -0.14  (0.07) | 0.013 | 0.02  (0.005) | 0.003 |
| Non-LPD  group | 31.25 ± 5.57 |  | 0 (-0.60 - 0.90) |  | -0.06  (0.06) |  | 0.06  (0.06) |  | -0.001  (0.004) |  |
| EWR |  |  |  |  |  |  |  |  |  |  |
| LPD  group | 0.39 (0.38 - 0.39) | 0.515^*^ | 0.0004  (-0.004 - 0.003) | 0.594^*^ | 0.00003  (0.0003) | 0.430 | -0.0004  (0.0003) | 0.177 | -0.0005  (0.0002) | 0.117 |
| Non-LPD  group | 0.39 (0.38 - 0.39) |  | 0.0009  (-0.003 - 0.002) |  | 0.0003  (0.0002) |  | 0.00003  (0.0002) |  | -0.0001  (0.0002) |  |
| WHR |  |  |  |  |  |  |  |  |  |  |
| LPD  group | 0.84 (0.81 - 0.88) | 0.218* | 0 (-0.02 - 0.02) | 0.143^*^ | 0.001  (0.002) | 0.026 | 0.003  (0.002) | 0.007 | 0.001  (0.001) | 0.250 |
| Non-LPD  group | 0.84 (0.80 - 0.87) |  | 0 (-0.01 - 0.03) |  | -0.004  (0.001) |  | -0.003  (0.002) |  | -0.0005  (0.001) |  |
| FFM (kg) |  |  |  |  |  |  |  |  |  |  |
| LPD  group | 43.39 ± 8.07 | 0.478^**^ | 0.75  (0 - 1.78) | 0.018^*^ | -0.36  (0.10) | 0.025 | -0.20  (0.09) | 0.008 | -0.02  (0.007) | 0.003 |
| Non-LPD  group | 42.44 ± 7.51 |  | 0.10  (-0.80 - 1.20) |  | -0.07  (0.08) |  | 0.10  (0.08) |  | 0.003  (0.006) |  |
| AC (cm) |  |  |  |  |  |  |  |  |  |  |
| LPD  group | 27.79 ± 2.53 | 0.149^**^ | 0.50  (-0.48 - 1.00) | 0.914^*^ | -0.14  (0.05) | 0.718 | 0.04  (0.03) | 0.067 | 0.005  (0.007) | 0.762 |
| Non-LPD  group | 28.49 ± 3.02 |  | 0.40  (-0.20 - 0.90) |  | -0.17  (0.05) |  | -0.03  (0.03) |  | 0.008  (0.006) |  |
| AMC (cm) |  |  |  |  |  |  |  |  |  |  |
| LPD  group | 24.53 ± 2.28 | 0.405^**^ | 0.35  (-0.18 - 0.78) | 0.777^*^ | -0.13  (0.04) | 0.979 | -0.02  (0.03) | 0.531 | -0.01  (0.008) | 0.993 |
| Non-LPD  group | 24.86 ± 2.36 |  | 0.30  (-0.20 - 0.80) |  | -0.13  (0.04) |  | -0.04  (0.03) |  | -0.01  (0.007) |  |

^a^Values are given as medians (interquartile range, IQR). *P*≤0.05 was considered statistically significant.

^b^Difference value between baseline and the fifth follow-up time point

^c^Mixed linear model of all data at five follow-up time points. Model Ⅰ adjusted for grouping, time, and interaction between group and time. Model Ⅱ adjusted for demographic data on the basis of Model Ⅰ [including age, sex, height, weight, standard body weight (SBW), body mass index (BMI), education level, retirement status, marital status, comorbidities, systolic blood pressure (SBP) and diastolic blood pressure (DBP)]. Model Ⅲ adjusted for all covariates in Model II and body composition analysis, dietary protein intake (DPI), dietary energy intake (DEI) and laboratory tests [including total body weight (TBW), extracellular water ratio (EWR), waist-hip ratio (WHR), fat free mass (FFM), arm circumference (AC), arm muscle circumference (AMC), serum creatinine (SCR), urea, serum uric acid (UA), carbon dioxide combining power (CO_2_CP), estimated glomerular filtration rate (eGFR), triglycerides (TG), total cholesterol (TC), albumin (ALB), hemoglobin (Hb) and urinary protein/creatinine ratio (UPCR)].

SE: standard error. ^*^Mann-Whitney U test. ^**^ *t*-test

Supplemental Table 4: Mixed linear model Ⅰ^a^

| **Variable** | **-2log-likelihood** | **Qualified Group**  **Slope (SE)** | **Unqualified Group**  **Slope (SE)** | **Difference**  **(95% CI limits)** | ***P* value** |
| --- | --- | --- | --- | --- | --- |
| Weight | 3,825.156 | -0.43 (0.13) | -0.29 (0.11) | -0.14 (-0.47, 0.20) | 0.433 |
| BMI | 2,318.249 | -0.32 (0.06) | -0.15 (0.05) | -0.16 (-0.31, -0.01) | 0.032 |
| SBP | 6,159.806 | 1.35 (0.78) | 0.97 (0.69) | 0.38 (-1.65, 2.42) | 0.711 |
| DBP | 5,631.259 | 0.33 (0.56) | 0.35 (0.50) | -0.02 (-1.49, 1.45) | 0.978 |
| UPCR | 1,770.090 | -0.21 (0.05) | 0.17 (0.04) | -0.38 (-0.51, -0.25) | ＜0.001 |
| TG | 1,801.974 | -0.12 (0.05) | 0.09 (0.05) | -0.21 (-0.36, -0.06) | 0.006 |
| TC | 1,865.049 | -0.12 (0.06) | 0.02 (0.06) | -0.13 (-0.30, 0.03) | 0.107 |
| Hb | 5,393.521 | -0.82 (0.63) | -1.50 (0.57) | 0.68 (-0.99, 2.34) | 0.424 |
| ALB | 3,637.125 | -0.74 (0.23) | -0.89 (0.21) | 0.21 (-0.45, 0.75) | 0.626 |
| SCR | 8,340.715 | 24.85 (5.61) | 29.47 (4.97) | -4.63 (-19.35, 10.09) | 0.537 |
| UREA | 3,910.052 | 0.95 (0.24) | 1.18 (0.21) | -0.22 (-0.84, 0.39) | 0.477 |
| UA | 8,042.007 | -10.98 (5.58) | -7.45 (4.94) | -3.53 (-18.16, 11.10) | 0.636 |
| TCO_2_ | 3,332.944 | -0.62 (0.18) | -0.66 (0.16) | 0.04 (-0.44, 0.52) | 0.868 |
| eGFR | 4,717.664 | -1.32 (0.37) | -2.35 (0.33) | 1.03 (-0.07, 2.00) | 0.036 |
| TBW | 2,644.314 | -0.25 (0.07) | -0.06 (0.06) | -0.20 (-0.38, -0.01) | 0.039 |
| WHR | -2,926.327 | 0.001 (0.002) | -0.004 (0.001) | -0.005 (-0.001, 0.009) | 0.026 |
| FFM | 3,060.885 | -0.36 (0.10) | -0.07 (0.08) | -0.29 (-0.54, -0.04) | 0.025 |
| EWR | -5,560.856 | 3.251242E^-5^  (0.0003) | 0.0003 (0.0002) | -0.0003  (-0.001, 0.0004) | 0.430 |
| AC | 2,082.192 | -0.14 (0.05) | -0.17 (0.05) | 0.03 (-0.11, 0.16) | 0.718 |
| AMC | 1,760.709 | -0.13 (0.04) | -0.13 (0.04) | -0.001 (-0.11, 0.11) | 0.979 |

^a^Mixed linear model of all data at five follow-up time points. *P*≤0.05 was considered statistically significant. Model I adjusted for grouping, time, and interactions between groups and time.

SE: standard error; CI: confidence interval; BMI: body mass index; SBP: systolic blood pressure; DBP: diastolic blood pressure; UPCR: urinary protein/creatinine ratio; TG: triglycerides; TC: total cholesterol; Hb: hemoglobin; ALB: albumin; SCR: serum creatinine; UA: serum uric acid; CO_2_CP: carbon dioxide combining power; eGFR: estimated glomerular filtration rate; TBW: total body weight; EWR: extracellular water ratio; WHR: waist-hip ratio; FFM: fat free mass; AC: arm circumference; AMC: arm muscle circumference

Supplemental Table 5: Mixed linear model II^a^

| **Variable** | **-2log-likelihood** | **Qualified Group**  **Slope (SE)** | **Unqualified Group**  **Slope (SE)** | **Difference**  **(95% CI limits)** | ***P* value** |
| --- | --- | --- | --- | --- | --- |
| Weight | 2,663.507 | 0.28 (0.10) | 0.004 (0.09) | 0.28 (0.03, 0.52) | 0.026 |
| BMI | 1,393.664 | -0.17 (0.04) | -0.04 (0.03) | -0.13 (-0.23, -0.03) | 0.013 |
| SBP | 5,405.945 | 1.13 (0.71) | 0.42 (0.63) | 0.70 (-1.11, 2.53) | 0.449 |
| DBP | 4,934.063 | -0.06 (0.51) | 0.29 (0.44) | -0.23 (-1.53, 1.06) | 0.722 |
| UPCR | 1,714.610 | -0.21 (0.05) | 0.14 (0.05) | -0.35 (-0.48, -0.22) | ＜0.001 |
| TG | 1,693.962 | -0.09 (0.06) | 0.08 (0.05) | -0.17 (-0.31, -0.02) | 0.024 |
| TC | 1,800.417 | -0.12 (0.06) | 0.02 (0.06) | -0.11 (-0.27, 0.06) | 0.196 |
| Hb | 5,077.282 | -0.91 (0.68) | -1.78 (0.62) | 0.87 (-0.81, 2.55) | 0.310 |
| ALB | 3,319.149 | -0.74 (0.20) | -0.74 (0.18) | 0.01 (-0.53, 0.51) | 0.982 |
| SCR | 7,840.310 | 29.32 (6.02) | 31.94 (5.40) | -2.62 (-17.73, 12.49) | 0.733 |
| UREA | 3,725.425 | 1.10 (0.25) | 1.28 (0.23) | -0.18 (-0.82, 0.46) | 0.583 |
| UA | 7,552.640 | -7.05 (5.57) | -6.14 (5.01) | -0.91 (-15.283, 13.46) | 0.901 |
| TCO_2_ | 3,187.310 | -0.70 (0.19) | -0.70 (0.17) | -0.002 (-0.48, 0.48) | 0.992 |
| eGFR | 4,488.863 | -1.90 (0.43) | -2.70 (0.39) | 0.80 (-0.20, 1.79) | 0.115 |
| TBW | 2,108.503 | -0.14 (0.07) | 0.06 (0.06) | -0.20 (-0.36, -0.43) | 0.013 |
| WHR | -2,890.845 | 0.003 (0.002) | -0.003 (0.002) | 0.006 (0.002, 0.01) | 0.007 |
| FFM | 2,515.272 | -0.20 (0.09) | 0.10 (0.08) | -0.30 (-0.51, -0.08) | 0.008 |
| EWR | -5,376.864 | -0.0004  (0.0003) | 2.693481E^-5^ (0.0002) | -0.0004  (-0.001, 0.0002) | 0.177 |
| AC | 1,036.283 | 0.04 (0.03) | -0.03 (0.03) | 0.07 (-0.004, 0.14) | 0.067 |
| AMC | 1,010.207 | -0.02 (0.03) | -0.04 (0.03) | 0.02 (-0.05, 0.10) | 0.531 |

^a^Mixed linear model of all data at five follow-up time points. *P*≤0.05 was considered statistically significant. Model II adjusted demographic data on the basis of Model I [including age, sex, height, weight, standard body weight (SBW), body mass index (BMI), education level, retirement state, marital status, comorbidities, systolic blood pressure (SBP) and diastolic blood pressure (DBP)].

SE: standard error; CI: confidence interval; UPCR: urinary protein/creatinine ratio; TG: triglycerides; TC: total cholesterol; Hb: hemoglobin; ALB: albumin; SCR: serum creatinine; UA: serum uric acid; CO_2_CP: carbon dioxide combining power; eGFR: estimated glomerular filtration rate; TBW: total body weight; EWR: extracellular water ratio; WHR: waist-hip ratio; FFM: fat free mass; AC: arm circumference; AMC: arm muscle circumference

Supplemental Table 6: Mixed linear model III^a^

| **Variable** | **-2log-likelihood** | **Qualified Group**  **Slope (SE)** | **Unqualified Group**  **Slope (SE)** | **Difference**  **(95% CI limits)** | ***P* value** |
| --- | --- | --- | --- | --- | --- |
| Weight | 1,156.307 | 0.01 (0.04) | -0.03 (0.03) | 0.04 (-0.05, 0.12) | 0.397 |
| BMI | 1,226.221 | -0.17 (0.05) | -0.04 (0.04) | -0.13 (-0.25, -0.01) | 0.032 |
| SBP | 4,481.834 | 1.61 (0.86) | -0.02 (0.72) | 1.58 (-0.48, 3.64) | 0.132 |
| DBP | 4,088.656 | 0.30 (0.60) | 0.48 (0.49) | -0.18 (-1.59, 1.23) | 0.803 |
| UPCR | 1,555.551 | -0.32 (0.06) | 0.003 (0.05) | -0.31 (-0.44, -0.18) | ＜0.001 |
| TG | 1,554.746 | -0.02 (0.06) | 0.09 (0.05) | -0.11 (-0.26, 0.03) | 0.122 |
| TC | 1,626.201 | -0.04 (0.07) | -0.01 (0.05) | -0.02 (-0.17, 0.13) | 0.759 |
| Hb | 4,361.664 | 2.15 (0.70) | 0.43 (0.59) | 1.72 (0.12, 3.33) | 0.035 |
| ALB | 3,031.680 | -1.24 (0.23) | -0.76 (0.19) | -0.48 (-1.03, 0.73) | 0.089 |
| SCR | 5,871.817 | 9.52 (2.74) | 3.88 (2.28) | 5.63 (-0.78, 12.05) | 0.085 |
| UREA | 2,801.060 | 0.12 (0.19) | 0.22 (0.16) | -0.10 (-0.55, 0.35) | 0.663 |
| UA | 6,847.964 | -13.56 (6.58) | -11.74 (5.43) | -1.82 (-17.29, 13.66) | 0.818 |
| TCO_2_ | 2,901.423 | -0.55 (0.21) | -0.54 (0.18) | 0.01 (-0.52, 0.50) | 0.974 |
| eGFR | 3,702.726 | 0.30 (0.39) | -0.14 (0.32) | 0.45 (-0.44, 1.33) | 0.325 |
| TBW | -1,326.926 | 0.02 (0.005) | -0.001 (0.004) | 0.02 (0.01, 0.03) | 0.003 |
| WHR | -2,588.983 | 0.001 (0.001) | -0.0005 (0.001) | 0.002 (-0.001, 0.004) | 0.250 |
| FFM | -975.274 | -0.02 (0.007) | 0.003 (0.006) | -0.02 (-0.04, -0.008) | 0.003 |
| EWR | -4,931.054 | -0.0005  (0.0002) | -0.0001  (0.0002) | -0.0004  (-0.0008, 0.9290597E^-5^) | 0.117 |
| AC | -802.416 | 0.005 (0.007) | 0.008 (0.006) | -0.002 (0.02, 0.01) | 0.762 |
| AMC | -706.897 | -0.01 (0.008) | -0.01 (0.007) | -8.086415E^-5^ (-0.02, 0.02) | 0.993 |

^a^Mixed linear model of all data at five follow-up time points. *P*≤0.05 was considered statistically significant. Model III adjusted for all covariates in Model II, as well as body composition analyses, dietary protein intake (DPI), dietary energy intake (DEI) and laboratory tests [including total body weight (TBW), extracellular water ratio (EWR), waist-hip ratio (WHR), fat free mass (FFM), arm circumference (AC), arm muscle circumference (AMC), serum creatinine (SCR), urea, serum uric acid (UA), carbon dioxide combining power (CO_2_CP), estimated glomerular filtration rate (eGFR), triglycerides (TG), total cholesterol (TC), albumin (ALB), hemoglobin (Hb) and urinary protein/creatinine ratio (UPCR)].

SE: standard error; CI: confidence interval; BMI: body mass index; SBP: systolic blood pressure; DBP: diastolic blood pressure

| **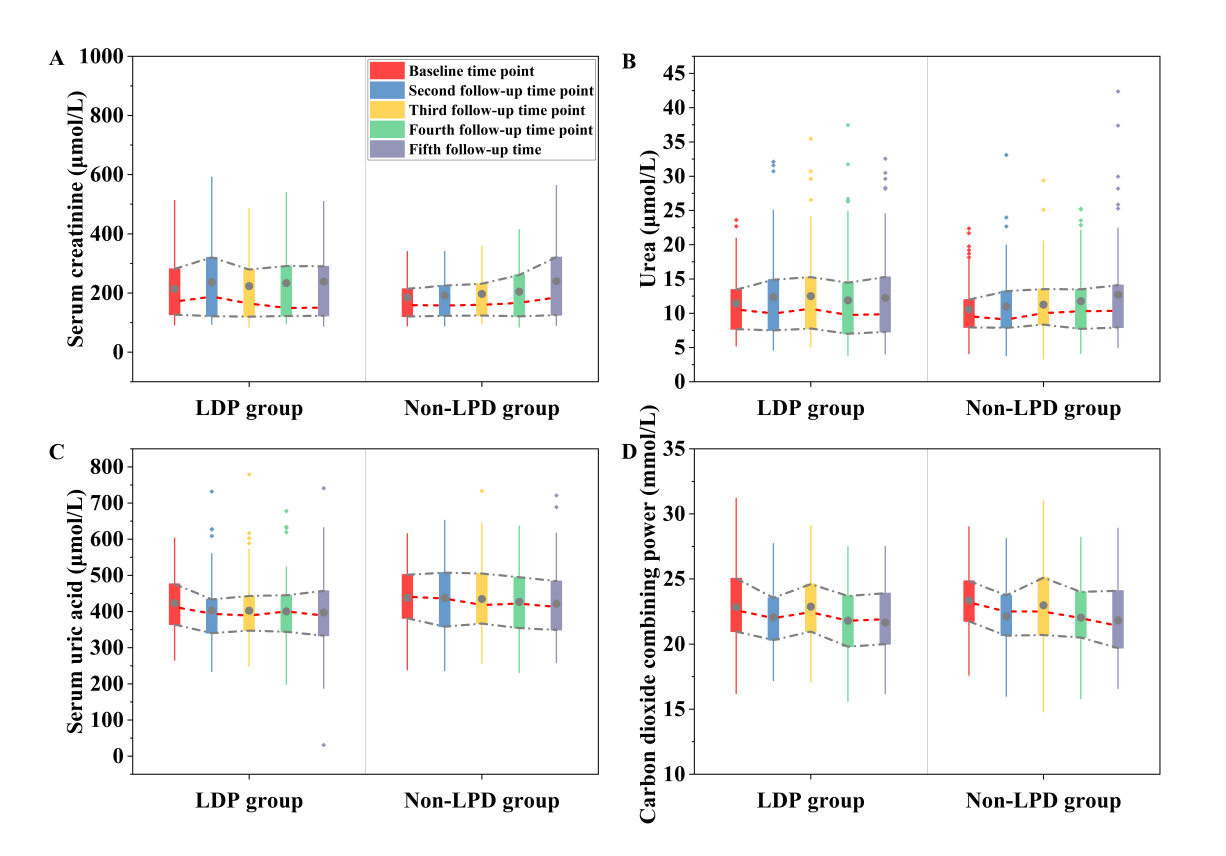**  **Supplemental Figure 1**  **Boxplot of renal function indicators at 5 follow-up time points**  Compared with the Non-LPD group, there was no significant upward trend of serum creatinine in the LPD group, and the trends of other renal function indicators were not significantly different between the two groups. |
| --- |

| **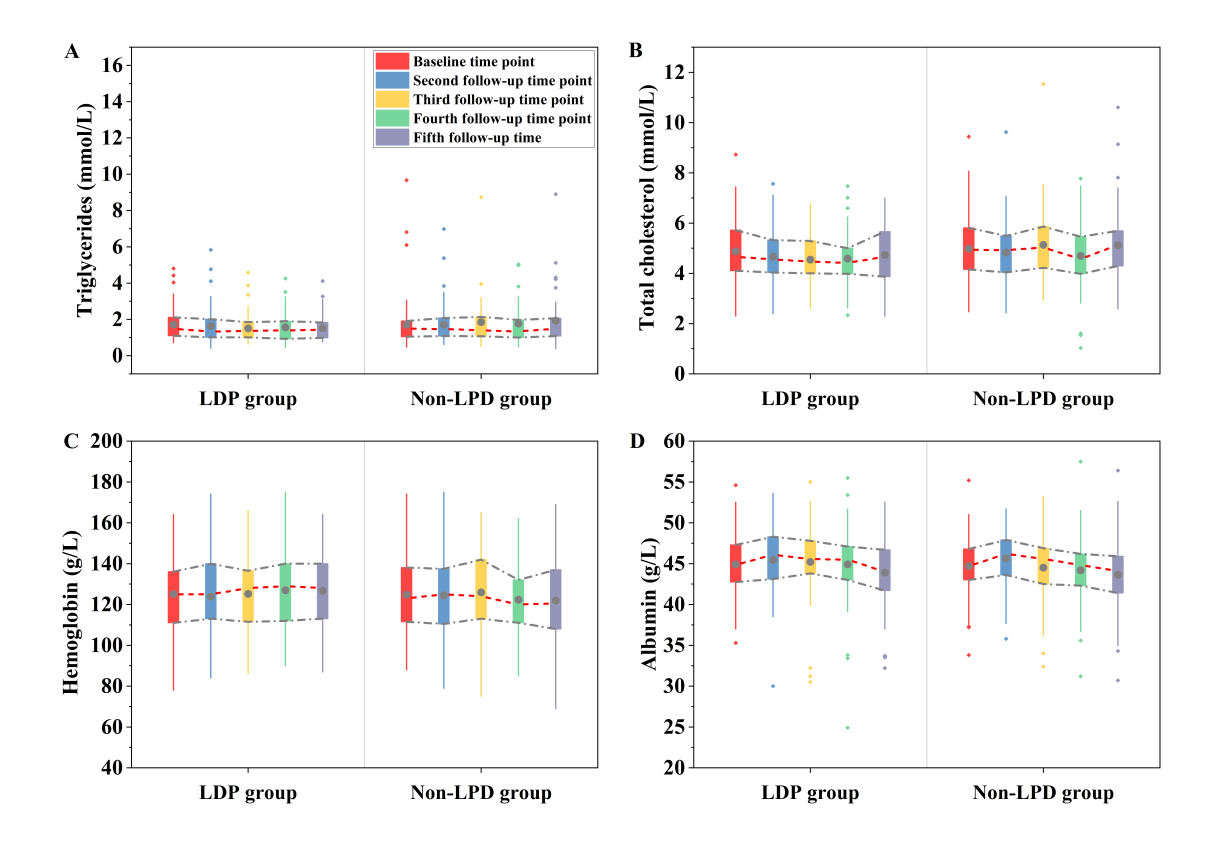**  **Supplemental Figure 2**  **Boxplot of indicators related to nutritional assessment at 5 follow-up time points (1)**  The trends of triglycerides were statistically significantly different in the LPD group compared with the Non-LPD group, and the remaining trends such as total cholesterol, hemoglobin, and albumin were similar in both groups. |
| --- |

| **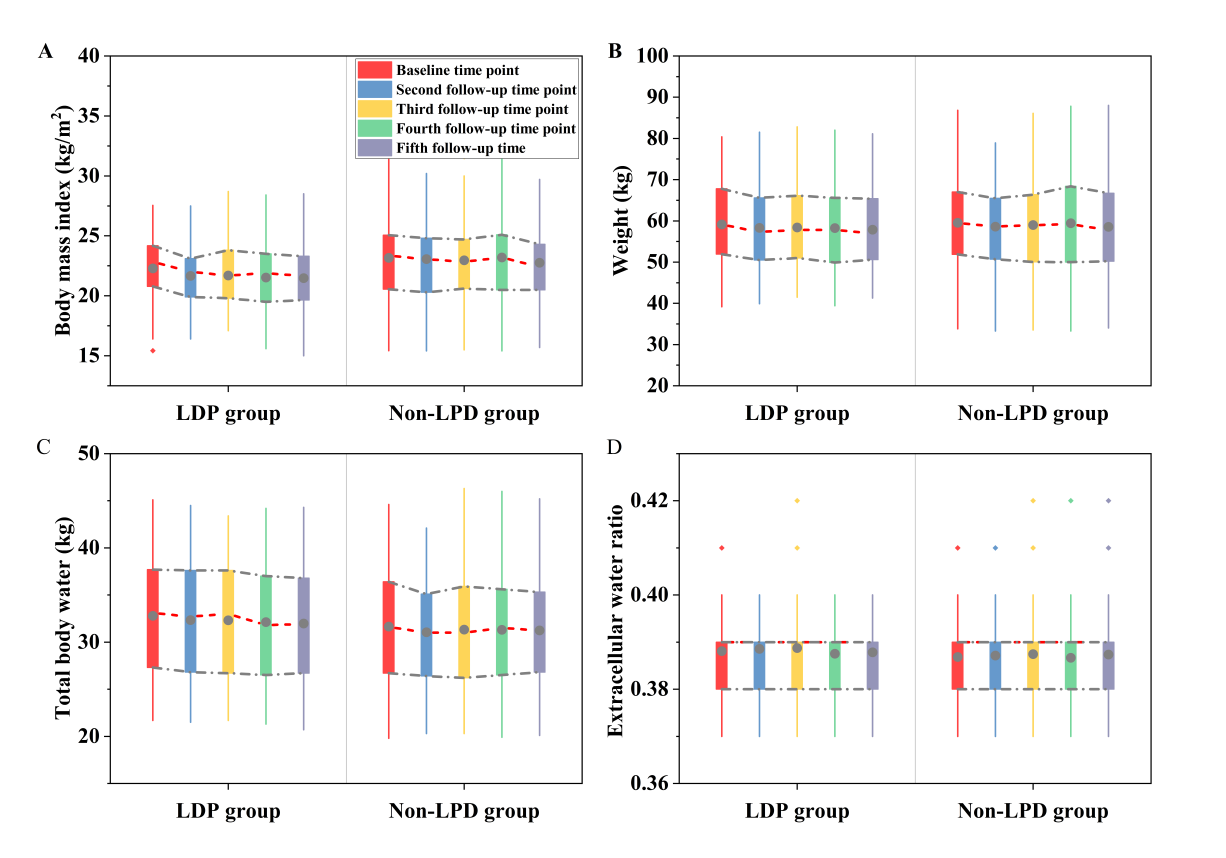**  **Supplemental Figure 3**  **Boxplot of indicators related to nutritional assessment at 5 follow-up time points (2)**  Boxplots depict trends in a subset of body composition analysis indicators. There is some degree of decline in body mass index, weight and total body water. |
| --- |

| **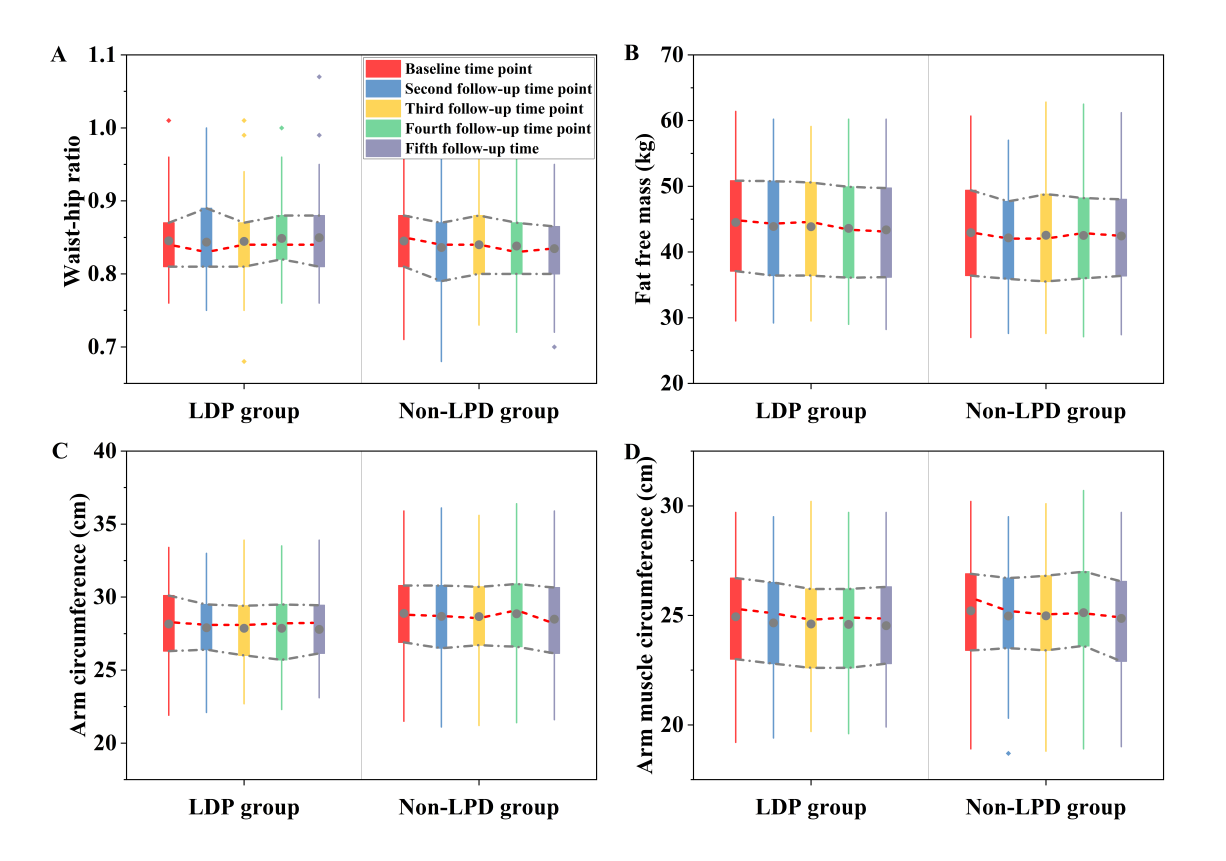**  **Supplemental Figure 4**  **Boxplot of indicators related to nutritional assessment at 5 follow-up time points (3)**  Boxplots depict trends in a subset of body composition analysis indicators. The changes of FFM in the LPD group showed a decreasing trend, while the differences in waist-to-hip ratio, arm circumference and arm muscle circumference were not significant when the two groups were compared. |
| --- |
